# Potent Bile Acid Microbial Metabolites Modulate *Clostridium perfringens* Virulence

**DOI:** 10.3390/pathogens12101202

**Published:** 2023-09-28

**Authors:** Tahrir Alenezi, Ying Fu, Bilal Alrubaye, Thamer Alanazi, Ayidh Almansour, Hong Wang, Xiaolun Sun

**Affiliations:** 1 Center of Excellence for Poultry Science, University of Arkansas, Fayetteville, AR 72701, USA; tjalenez@uark.edu (T.A.); yingfu@uark.edu (Y.F.); alrubaye@uark.edu (B.A.); alanazi@uark.edu (T.A.); amamansour@sfda.gov.sa (A.A.); hxw01@uark.edu (H.W.); 2 Cell and Molecular Biology, University of Arkansas, Fayetteville, AR 72701, USA; 3 College of Medical Applied Sciences, The Northern Border University, Arar 91431, Saudi Arabia

**Keywords:** bile acid, necrotic enteritis, *C. perfringens*, toxic gas

## Abstract

*Clostridium perfringens* is a versatile pathogen, inducing diseases in the skin, intestine (such as chicken necrotic enteritis (NE)), and other organs. The classical sign of NE is the foul smell gas in the ballooned small intestine. We hypothesized that deoxycholic acid (DCA) reduced NE by inhibiting *C. perfringens* virulence signaling pathways. To evaluate the hypothesis, *C. perfringens* strains CP1 and wild-type (WT) HN13 and its mutants were cultured with different bile acids, including DCA and isoallolithocholic acid (isoalloLCA). Growth, hydrogen sulfide (H_2_S) production, and virulence gene expression were measured. Notably, isoalloLCA was more potent in reducing growth, H_2_S production, and virulence gene expression in CP1 and WT HN13 compared to DCA, while other bile acids were less potent compared to DCA. Interestingly, there was a slightly different impact between DCA and isoalloLCA on the growth, H_2_S production, and virulence gene expression in the three HN13 mutants, suggesting possibly different signaling pathways modulated by the two bile acids. In conclusion, DCA and isoalloLCA reduced *C. perfringens* virulence by transcriptionally modulating the pathogen signaling pathways. The findings could be used to design new strategies to prevent and treat *C. perfringens*-induced diseases.

## 1. Introduction

*Clostridium perfringens* is a spore-forming, anaerobic, and Gram-positive bacterium and is responsible for a variety of acute diseases, such as chicken necrotic enteritis (NE), foodborne enteritis, and myonecrosis [1]. It is estimated that NE causes a USD 6 billion loss to the poultry industry worldwide every year [2]. The increased NE incidence mainly resulted from the ongoing restriction of prophylactic antimicrobial usage in poultry production, while no effective alternatives are available [3]. Infection of coccidia of *Eimeria* (e.g., *E. maxima* and *acervuline*) is one of the prevalent predisposed factors in poultry farms [4]. Birds with severe clinical NE are shown with severe diarrhea, foul-smell and distended small intestine, mortality, anorexia, and reduced body weight gain and feed efficiency [5].

*C. perfringens* strains are classified into seven toxin types (A to G) based on their ability to produce the various combinations of six major toxins, namely alpha (CPA), beta (CPB), epsilon (ETX), iota (ITX), enterotoxin (CPE), and enteritis B-like toxin (NetB) [1,6,7]. Various combinations of the toxins cause necrosis of muscle, subcutaneous, and mucosal tissue in addition to the production of toxic gas of hydrogen sulfide (H_2_S) [1]. Most *C. perfringens* isolates produce α-toxin (CPA or PLC) encoded by *the plc (*or *cpa)* gene [8]. In the progression of gas gangrene, CPA penetrates the cell membrane with holes and activates arachidonic acid cascade and protein kinase C, resulting in cellular dysfunction [8,9]. In addition, CPA suppresses the immune response by restraining leukocytes entering the infected tissues [10] and reduces the blood supply to infected sites by triggering vasoconstriction, thrombosis, and platelet aggregation [8]. The gene expression of *plc*, perfringolysin O (*pfoA*), κ-toxin (*colA*), alpha-clostripain(*ccp*), and some extracellular enzymes is regulated by the VirS/VirR system [11,12], small regulatory RNA molecules (sRNAs: *vrr, virT,* and *virU*), or the RevR orphan response regulator [13]. *virT* gene expression is negatively correlated to *pfoA* and ccp, whereas the *virU* gene product positively affects the expression of *pfoA*, *virT*, *ccp*, and *vrr* [14].

Another toxin of *C. perfringens* enterotoxin (CPE) encoded by the *cpe* gene is a 35.5 kDa polypeptide [15] and is associated with chicken NE [16]. This toxin is only expressed during *C. perfringens* sporulation. It is accumulated at the beginning of the sporulation and released after cell lysis at the end of the sporulation [17,18]. CPE production is tightly controlled through the master sporulation regulator Spo0A, the transcriptional factor NanR, and three sigma factors of SigE, SigF, and SigK [19,20]. Spo0A mediates sporulation and biofilm formation through phosphorylation and orphan histidine kinases [21,22,23]. H_2_S is a volatile gas with a foul odor of rotten eggs that is enzymatically produced by both eukaryotic and prokaryotic cells. H_2_S is a signaling molecule and modulates animal cellular processes, including cell proliferation, apoptosis, inflammation, and hypoxia [24,25,26]. H_2_S in bacteria is produced during sulfur amino acid (methionine, cysteine, homocysteine, and taurine) metabolism. Methionine mediates methylation and polyamine biosynthesis for sadenosylmethionine (SAM) and quorum sensing autoinducer 2 (AI-2) [27]. Sulfite (e.g., taurine) is converted into sulfide (e.g., H_2_S) by anaerobic sulfite reductases (*asrABC1* (*cpe1438-1440*) and *asrABC2* (*cpe1536-1538*)) in *C. perfringens* [28].

Two main categories of primary bile acids are in the animal gastrointestinal tract: cholic acid (CA) and chenodeoxycholic acid (CDCA). The two primary bile acids are sequentially transformed to their respective bile acid lineages of intermediate bile and secondary bile acids of deoxycholic acid (DCA) and lithocholic acid (LCA), respectively [29,30]. The bile acids are also transformed by intestinal microbiota into 3/7/12-oxo-, iso-, -oxoallo-, isoallo-, or allo-forms such as isoallolithocholic acid (isoalloLCA) [30]. Because of the amphipathic property of bile acids, the physiological action of bile acids on cells is traditionally assumed through the non-selective detergent activity of disturbing the permeabilization of the cellular lipid membrane. Recent research progression has shed insight into specific signaling pathways of bile acids in cells. G-protein-coupled BA receptor (TGR5) is activated by both conjugated and unconjugated bile acids at the decreasing potency: LCA > DCA > CDCA > CA [31,32]. TGR5 activated by bile acids induces a cascade of signaling pathways of cAMP, PKA, or the exchange protein directly activated by cAMP (EPAC) [32,33,34]. Bile acids are the natural ligands of the nuclear receptor of farnesoid X receptor (FXR) with activation potency of CDCA > CA > DCA and UDCA [35], while LCA is an antagonist [36]. On the microorganism side, bile acids induce c-di-AMP degradation in *C. difficile*, which derepresses genes encoding a solute import system protecting from hyperosmotic stress and bile salts [37]. Bile acids are essential for in vitro *C. difficile* germination [38] and signal through the *csp*C germinant receptor with TCA the highest potency [39]. *C. perfringens* deconjugates conjugated bile acids [40] and transforms CDCA > CA into three beta isoforms [41]. Various bile acids also mediate *C. perfringens* growth, sporulation, and germination [42,43,44,45]. In a previous study [46], we found that only DCA significantly reduced *C. perfringens* strain CP1-induced chicken NE with improved growth performance and histopathology compared to ox bile (TCA and GCA), chicken bile (TCDCA and TCA), and LCA. In this study, we aimed to investigate how bile acids influenced *C. perfringens* virulence signaling pathways.

## 2. Materials and Methods

### 2.1. Strains, Media, Growth Condition, and Mariner-Based Transposon Mutagenesis

*C. perfringens* strain CP1, isolated from our previous chicken studies, was confirmed positive for various toxins, including *cpa*, *cpe*, and *netB* [1,3]. Bile acids CA, CDCA, DCA, and LCA were purchased from VWR (all from Alfa Aesar), while isoalloLCA was purchased from Cayman Chemical (Ann Arbor, Michigan, USA). The *C. perfringens* strains were grown in Brain Heart Infusion (BHI, Legacy biologicals, Mt Prospect, IL, USA) broth for preparing inoculum under anaerobic conditions for 24 h at 37 °C.

The *C. perfringens* strain HN13 generated at Dr. Nariya’s lab [47] and mariner-based transposon mutagenesis plasmid pHLL24 were kindly provided by Dr. Melville [48] at Virginia Tech. To generate a transposon mutant library following the reported procedures [48], briefly, pHLL24 was transformed to HN13 by electroporation. The resultant HN13 was selected on a BHI plate supplemented with 20 μg/mL chloramphenicol and 30 μg/mL erythromycin. The cells on the plate were collected, inoculated in BHI broth with the same antibiotic concentration, and grown to the mid-log phase. The cells were washed twice with PBS and cultured in BHI broth with 1 mM of lactose. After the 2 h culture, the cells were washed 3 times with PBS and incubated at 37 °C after 100-fold dilution in BHI. After 4 h, the 2XYT broth (VWR, Solon, OH, USA) with 30 μg/mL erythromycin and 3% galactose was used to dilute the previous culture at 100-fold, and the cells were incubated. After 4 h, the cells were plated on a 2XYT plate with galactose and erythromycin. Because colonies resisting galactose and erythromycin had transposon mutagenesis in the genome, the cells were collected in 20% glycerol and stored at −80 °C as HN13 transposon mutant library. To further select mutants resistant to DCA, the HN13 mutant library was inoculated in BHI broth with 3 mM DCA overnight. The cells were serially diluted and plated on 2XYT plates with galactose and erythromycin. Individual colonies from diluted plates or collective cells (non-diluted plate) were collected and stored at −80 °C. In this study, three HN13 mutants resistant to DCA were randomly selected for use.

### 2.2. Bile Acid Inhibition of C. perfringens Growth Assay

*C. perfringens* CP1 and HN13 at around 3.9 log10 colony forming unit (CFU) were inoculated into 1 mL TSB in the presence of CA, CDCA, DCA, LCA, and isoalloLCA at 0, 0.001, 0.01, 0.5, or 1 mM, and cultured for 24 h under anaerobic conditions. The bacterial growth was measured by CFU enumeration. To count CFU, the cells were serially diluted and plated on Tryptic Soy Agar (TSA, Criterion, Santa Maria, CA, USA) with sodium thioglycolate, D-Cycloserine, and sheep blood. Coculturing HN13 mutants with bile was similar to that of wild-type cells, except that the mutant cells were plated on 2XYT plates with galactose and erythromycin. Because of the precipitation of bile in cultured broth, the OD_600_ results were not consistent with the actual *C. perfringens* growth, and we only reported the CFU results.

### 2.3. Bacterial RNA Extraction and Gene Expression Using Real-Time PCR

*C. perfringens* CP1, HN13, or HN13 mutants were inoculated on BHI broth in the presence of DCA or isoalloLCA at 0, 0.01, 0.5, or 1 mM and were cultured under anaerobic conditions. After 2 h, the cells were collected, and total RNA was extracted using the TRIzol (ThermoFisher, St. Louis, MO, USA) as described before [16,49]. After cDNA was synthesized using M-MLV (NE Biolab, Ipswich, MA, USA) and random hexamer [50], the virulence gene expression (*asrA1*, *plc*, *spoOA*, *virT*, and *cpe*) was determined using SYBR Green PCR Master mix (Bio-Rad Hercules, CA, USA) on a Bio-Rad 384-well real-time PCR system as described before [46]. The primer sequences are shown in Table 1. The gene expression of the fold-change was calculated using the ΔΔCt method [50] and *gyrA* as an internal control.

### 2.4. H_2_S Detection Using Lead Acetate Assay

*C. perfringens* CP1, HN13, or HN13 mutants were cultured on BHI with bile acids at 0, 0.01, 0.5, and 1 mM. Filter papers, saturated with lead acetate (Pb(OAc)₂), were dried, autoclaved, and placed on the cap of the tube. The bacteria were cultured under anaerobic conditions at 37 °C. After 24 h, H_2_S production was visualized and imaged. H_2_S was produced when the paper changed from white to brown color. The darker the color, the more H_2_S production. The culture medium also smelled like rotten eggs.

### 2.5. Statistical Analysis

**Figure 1:**
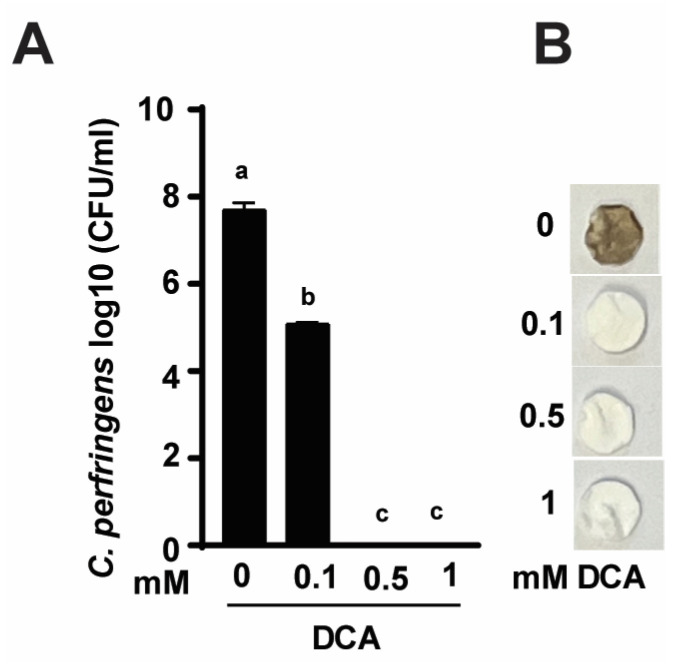
DCA reduces *C. perfringens* strain CP1 growth and H_2_S production. *C. perfringens* was cultured with DCA for 24 h under anaerobic conditions. (**A**) *C. perfringens* enumeration by serial dilution and plating. (**B**) Detection of H_2_S production via the lead acetate (Pb(OAc)₂) discs. Results are representative of 3 independent experiments. All graphs show mean + SEM. Different letters of a–c mean *p* < 0.05.

**Figure 2:**
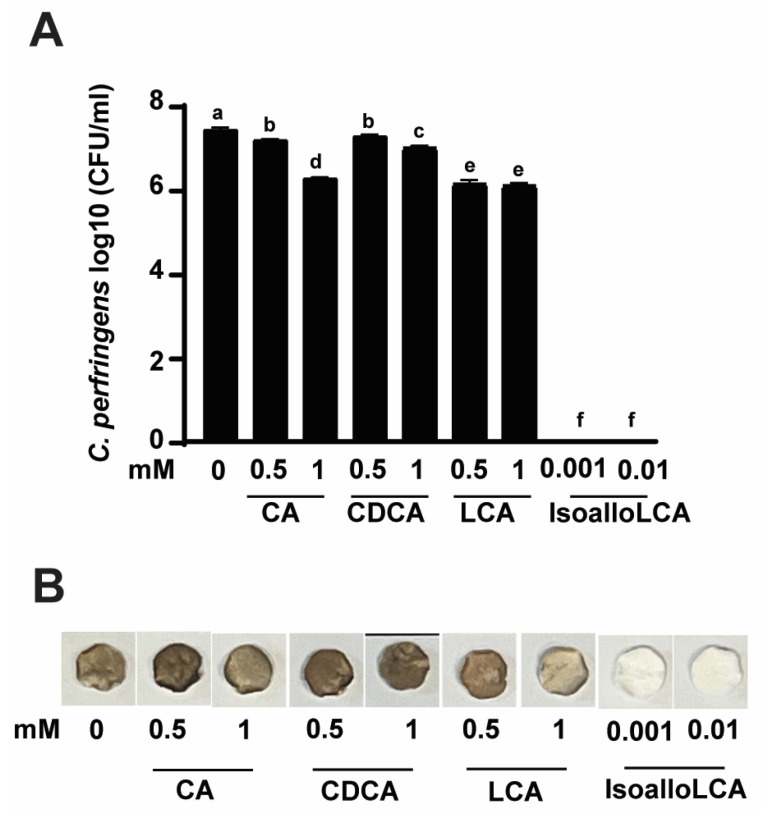
IsoalloLCA reduces *C. perfringens* strain CP1 growth and H_2_S production. *C. perfringens* was cultured with CA, CDCA, LCA, or isoalloLCA for 24 h under anaerobic conditions. (**A**) *C. perfringens* enumeration by serial dilution and plating. (**B**) Detection of H_2_S production via the lead acetate discs. Results are representative of 3 independent experiments. All graphs show mean + SEM. Different letters of a–f mean *p* < 0.05.

**Figure 3:**
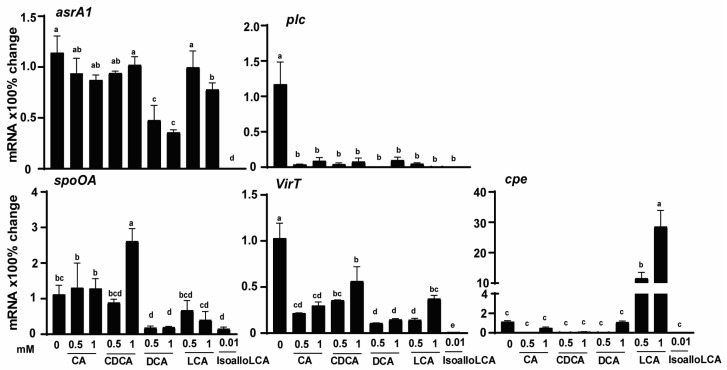
*C. perfringens* CP1 virulence gene expression is reduced by DCA and isoalloLCA. *C. perfringens* CP1 was cultured with CA, CDCA, DCA, LCA, and isoalloLCA for 2 h. After RNA extraction and cDNA reverse transcription, the virulence gene accumulation was quantified by real-time PCR (qPCR). Results are representative of 3 independent experiments. All graphs show mean + SEM. Different letters of a–e mean *p* < 0.05.

**Figure 4:**
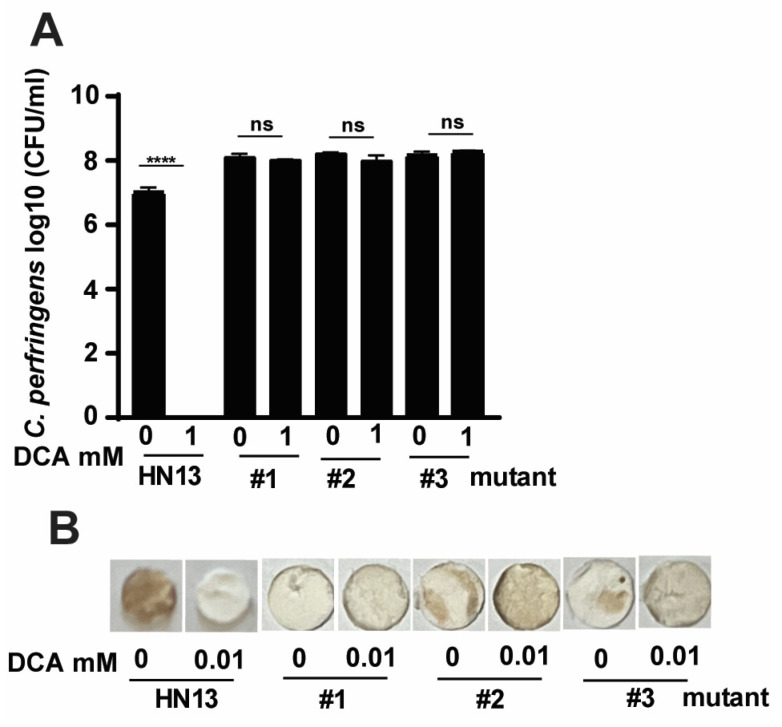
The effects of DCA on *C. perfringens* HN13 and its mutant growth and H_2_S production. *C. perfringens* strain HN13 and its three mutants were cultured with DCA for 24 h under anaerobic conditions. (**A**) *C. perfringens* enumeration by serial dilution and plating. (**B**) Detection of H_2_S production via the lead acetate discs. # means No. All graphs show mean + SEM. ****, *p* < 0.0001. ns, not significant.

**Figure 5:**
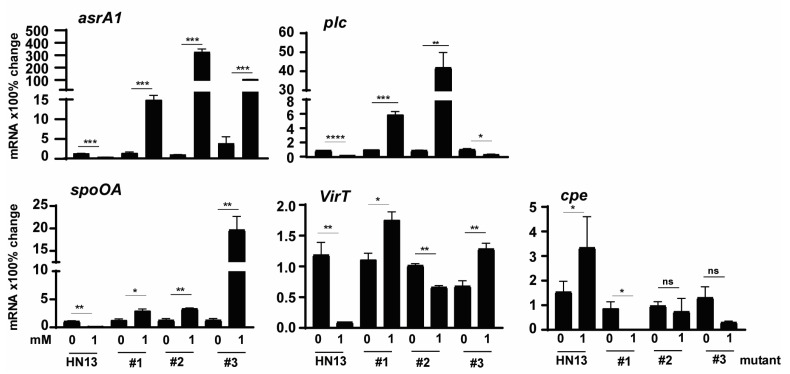
The effect of DCA on *C. perfringens* HN13 mutant virulence gene expression. *C. perfringens* HN13 and its three mutants were cultured with DCA for 2 h. After RNA extraction and cDNA reverse transcription, the virulence gene accumulation was quantified by real-time PCR (qPCR). Results are representative of 3 independent experiments. # means No. All graphs show mean + SEM. *, *p* < 0.05; **, *p* < 0.01; ***, *p* < 0.001; ****, *p* < 0.0001. ns, not significant.

**Figure 6:**
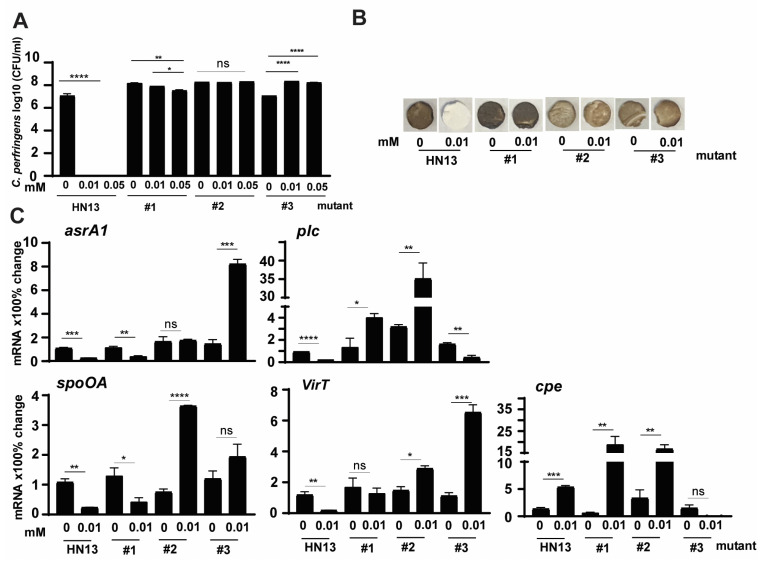
The effects of isoalloLCA on *C. perfringens* HN13 mutant growth, H_2_S production, and the virulence gene expression. (**A**) *C. perfringens* strain HN13 and its three mutants were cultured with isoalloLCA for 24 h under anaerobic conditions. *C. perfringens* enumeration by serial dilution and plating. (**B**) *C. perfringens* strain HN13 and its three mutants were cultured with isoalloLCA for 24 h under anaerobic conditions. Detection of H_2_S production via the lead acetate discs. (**C**) *C. perfringens* HN13 and its three mutants were cultured with isoalloLCA for 2 h. After RNA extraction and cDNA reverse transcription, the virulence gene accumulation was quantified by real-time PCR (qPCR). Results are representative of 3 independent experiments. # means No. All graphs show mean + SEM. *, *p* < 0.05; **, *p* < 0.01; ***, *p* < 0.001; ****, *p* < 0.0001. ns, not significant.

In Figure 1, Figure 2 and Figure 3, we used one-way ANOVA to compare means among multiple groups of bile treatments, all of which shared the same control group. Multiple comparisons of Fisher’s LSD test using Prism 7.0 software were then conducted. In Figure 4, Figure 5 and Figure 6, we used an unpaired *t*-test with Welch’s correction using Prism 7.0 software to compare means between two bile dose groups in either WT HN13 or its individual mutants, each of which had its own control group. Experiments were considered statistically significant if *p*-values were <0.05.

## 3. Results

### 3.1. DCA Reduces C. perfringens Strain CP1 Growth and H_2_S Production

To investigate the mechanism of DCA-reduced *C. perfringens* CP1-induced NE [46], we conducted an in vitro DCA growth inhibition assay. As shown in Figure 1A, *C. perfringens* growth reached 7.7 log10 CFU/mL after 24 h culture. Notably, DCA at 0.1, 0.5-, and 1-mM reduced *C. perfringens* by 33.9 (2.6 log10 CFU/mL), 100 (7.7 log10 CFU/mL), and 100% (7.7 log10 CFU/mL), respectively. At the clinical levels, DCA reduced severe diarrhea and small intestine ballooned with rotten egg-smelling gas in NE birds. We hypothesized that DCA might reduce *C. perfringens*-produced foul-smell H_2_S. Using H_2_S assay, *C. perfringens* culture had a rotten egg smell and turned disc paper brown (Figure 1B). Consistent with growth inhibition, DCA reduced the foul smell and the dark brown color of the discs in a dose-dependent manner.

### 3.2. IsoalloLCA Reduces C. perfringens CP1 Growth and H_2_S Production More Effectively Compared to Other Bile Acids

Encouraged by the finding of DCA inhibiting *C. perfringens* growth and H_2_S production, we then conducted a growth inhibition assay using the rest of the primary and secondary bile acids with an addition of isoalloLCA, reported as highly potent against *C. perfringens* growth [30]. As shown in Figure 2A, *C. perfringens* CP1 growth reached 7.38 log10 CFU after 24 h culture. Interestingly, CA or CDCA at 0.5 vs. 1 mM reduced *C. perfringens* growth by 3.38 vs. 15.71 (0.25 vs. 1.16 log10 CFU/mL) or 2.16 vs. 5.6% (0.16 vs. 0.41 log10 CFU/mL), respectively. Interestingly, LCA at 0.5 vs. 1 mM comparably reduced the pathogen growth by 17.3% (1.28 log10 CFU/mL). Notably, isoalloLCA at 0.001 and 0.01 mM completely (100%) reduced *C. perfringens* growth, suggesting its high potency compared to other bile acids. Consistent with the growth inhibition assay, H_2_S produced from *C. perfringens* was inhibited by isoalloLCA but barely by CA, CDCA, and LCA (Figure 2B).

### 3.3. Bile Acids Reduce C. perfringens CP1 Virulence Gene Expression

The reduction of H_2_S production by DCA and isoalloLCA could be mediated at a transcriptional or translational level. Because of the lack of antibodies against most *C. perfringens* proteins, it was more feasible to measure its transcription vs. translational activities. In addition, we reasoned that the bile acids would modulate other *C. perfringens* virulent activities. Hence, *C. perfringens* was cultured with different bile acids, and its RNA was extracted. Gene expressions of virulence genes were measured using real-time PCR. Consistent with the Pb(OAc)_2_ disc assay, the expression of the H_2_S-producing gene *asrA1* was reduced by 58, 69, and 100% with 0.5 mM DCA, 1 mM DCA, and 0.01 mM isoalloLCA, respectively, while 1 mM LCA reduced *asrA1* by 32% (Figure 3). Notably, toxin A gene *plc* accumulation was reduced universally across all bile at more than 91%. Master regulatory gene *spoOA* was reduced by 0.5 (84%) and 1 mM (83%) DCA, 1 mM LCA (64%), and 0.01 mM isoalloLCA (86%), while 1 mM CDCA increased the gene by 134%. The regulatory RNA gene *virT* was reduced by all bile from 45 to 99%, with CDCA and isoalloLCA being the least and most potent ones, respectively. Interestingly, 0.5 and 1 mM LCA increased enterotoxin gene *cpe* expression by 1157 and 2856%, respectively. These results suggest that bile differentially modulated the H_2_S production and other virulence activities, at least at the transcriptional level.

### 3.4. C. perfringens HN13 Mutants Produce H_2_S in the Presence of DCA

To further confirm that *C. perfringens* virulence modulated by bile was through specific signaling pathways, we sought to investigate the molecular signaling using *C. perfringens* strain HN13 and mariner-based transposon mutagenesis mutants [48]. The generated mutants were then selected for bile resistance using 3 M DCA, in which concentration no WT HN13 could grow. Among the thousands of mutant colonies, we randomly picked three colonies for the following assays. Consistent with *C. perfringens* strain CP1, the growth of wild-type strain HN13 was completely (100%) reduced by 1 mM DCA (Figure 4A). Notably, the growth rates of HN13 mutants #1, #2, and #3 were not changed by 1 mM DCA. We then examined the H_2_S production in the mutants using Pb(OAc)_2_ disc assay. As shown in Figure 4B, H_2_S produced in WT HN13 was greatly reduced by 1 mM DCA, an effect comparable to chicken isolate CP1. Interestingly, H_2_S production in the mutants was not inhibited but slightly increased by 1 mM DCA, suggesting that DCA modulates *C. perfringens* signaling pathways related to H_2_S production.

### 3.5. C. perfringens HN13 Mutants Express Virulence Genes in the Presence of DCA

To examine whether the modulation in the mutants was at the transcriptional level, we performed a real-time PCR assay. Notably, *C. perfringens* mutant isolates increased the expression of H_2_S production *asrA1* gene by 1012, 28, 115, and 805%, respectively, in the presence of 1 mM DCA (Figure 5), while WT HN13 reduced the gene expression by 73% (Figure 5). Notably, *plc* gene expression in mutants #1 and #2 increased by 469 and 4471% in the presence of DCA, while the gene expression in WT HN13 and mutant #3 reduced by 77 and 68%, respectively. Interestingly, *spoOA* gene expression in the mutants of # 1, #2, and #3 increased by 133, 164, and 1443%, respectively, in the presence of 1 mM DCA, while the gene reduced by 88% in WT HN13. The gene expression of *virT* in WT HN13 and mutant #2 reduced by 92 and 35%, respectively, in the presence of 1 mM DCA, while the gene accumulation increased in the mutants of #1 and #3 by 58 and 90%. In addition, 1 mM DCA increased *cpe* gene expression in WT HN13 by 117%, while the gene expression in mutants #1, #2, and #3 reduced by 100, 24, and 77%, respectively.

### 3.6. Effects of IsoalloLCA on Growth and Virulence of C. perfringens HN13 Mutants

Based on the results in Figure 1 and Figure 2, isoalloLCA was more potent than DCA in reducing *C. perfringens* CP1 growth and H_2_S production. To examine whether isoalloLCA modulated *C. perfringens* through the same signaling pathways as DCA, we investigated isoalloLCA impacts on WT HN13 and its three mutants. Consistent with the growth reduction by 1 mM DCA in Figure 4A, isoalloLCA at 0.01 and 0.05 mM completely inhibited WT HN13 growth (Figure 6A). Interestingly, the growth of mutant #1 was reduced by isoalloLCA, while isoalloLCA increased the growth of mutant #3. Notably, H_2_S production in WT HN13 was reduced by 0.01 mM isoalloLCA, while the gas produced by the three mutants was not reduced by the bile (Figure 6B). We then measured the change of *C. perfringens* virulence gene expression by 0.01 mM isoalloLCA. Similar to virulence gene reduction by 1 mM DCA in Figure 5, 0.01 mM isoalloLCA reduced the gene expression of *asrA1*, *plc*, *spoOA*, and *virT* in WT HN13 by 78, 80, 78, and 85%, respectively, while the bile increased *cpe* gene accumulation by 300% (Figure 6C). Interestingly, isoalloLCA reduced *asrA1* gene expression in mutant #1 by 67%, while the bile increased the gene expression in mutant #3 by 468%. IsoalloLCA increased *plc* gene expression by 200 and 1005% in mutants #1 and #2, respectively, while the bile reduced the gene expression in mutant #3 by 74%. IsoalloLCA reduced *spoOA* gene expression by 68% in mutant #1, while the bile increased the gene expression in mutant #2 by 384%. IsoalloLCA increased *virT* gene expression by 95 and 482% in mutants #2 and #3. IsoalloLCA increased *cpe* gene expression by 3033 and 408% in mutants #1 and #2, respectively.

## 4. Discussion

Dietary DCA effectively reduces *C. perfringens*-induced clinical [45,46] and subclinical chicken NE [49], but the molecular mechanism of DCA against *C. perfringens* remains largely elusive. In this study, we reasoned that bile acids were signaling pathway regulators modulating *C. perfringens* virulence activities. We found that isoalloLCA and DCA were more potent inhibitors toward *C. perfringens* growth, H_2_S production, and virulence gene expression compared to CA, CDCA, and LCA. The inhibition of virulence gene expression by the bile indicates regulation, at least at the transcriptional level. Based on the differential impact of DCA and isoalloLCA on the virulence of the three *C. perfringens* HN13 mutants, the two bile acids might act on slightly different signaling pathways. Together, these results suggest that bile acids regulated *C. perfringens* virulence signaling pathways with isoalloLCA and DCA as the most potent ones.

Our study results are consistent with the notion that bile acids are signaling regulators. Because bile acid has amphipathic detergent properties, it has traditionally been thought that the cellular toxicity of bile acids was mainly through non-selective detergent-like properties that disturb cellular membranes [51]. That concept was often discrepant with the observations that different bile acids had quite different or even opposite effects on cellular activities despite their comparable detergent properties. Research on animal molecular and cellular activities has revealed that bile acids regulate specific signaling pathways in embryogenesis, development, metabolism, and immunity [52]. G-protein-coupled bile acid receptor (TGR5) is activated by both conjugated and unconjugated bile acids at the decreasing potency of LCA > DCA > CDCA > CA [31,32]. TGR5 activated by bile acids induces a cascade of signaling pathways of cAMP, PKA, or the exchange protein directly activated by cAMP (EPAC) [32,33,34]. Bile acids also directly activate three nuclear receptors: FXR [53], pregnane X receptor (PXR) [54], and vitamin D receptor (VDR) [55]. FXR is activated at the decreasing potency of CDCA > LCA > DCA > CA, while LCA and 3-oxo-LCA activate VDR and FXR [55,56]. Furthermore, bile acids modulate host immunity. 3-oxoLCA inhibits Th17 cell differentiation by binding to retinoid-related orphan receptor-γt (RORγt), and isoalloLCA increases Treg cell differentiation through elevating mitochondrial reactive oxygen species (mitoROS), leading to FOXP3 expression increase [57]. IsoDCA increases Foxp3 expression by reducing FXR-related signaling pathways [58]. These findings highlight the important signaling regulatory role of bile acids on animals. It would be necessary to investigate the role of bile acids on chicken signaling pathways during NE in the future.

Because of the diverse composition of the bacterial community, the effect of bile acids on bacteria is highly variable between bacteria. Bile salts (mainly CA) have been used as the selective ingredient of bacterial growth medium to grow Gram-negative bacteria, such as MacConkey [59]. In this study, only DCA and isoalloLCA effectively inhibited the growth of the Gram-positive bacterium *C. perfringens*. The inhibition was in the decreasing potency of isoalloLCA > DCA > LCA > CA > CDCA. Because CDCA is the main bile acid of chickens [49], it is not surprising that chickens are susceptible to *C. perfringens*-induced NE. Besides mediating growth, bile acids also regulate *C. perfringens* sporulation and germination [42,43,44,45]. Similarly, DCA at 0.01% reduced the growth of another Gram-positive bacterium *C. difficile* [60], while CA at 2.4 mM promoted *C. difficile* spore germination but inhibited vegetative growth at 12 mM [61]. Interestingly, CDCA inhibits its spore germination and is a competitive inhibitor of germinant TCA [62]. The *csp*BAC locus is a major regulator of *C. difficile* germination, and *csp*C is the receptor for bile salt (T/GCA, CA) germinants, whereas the related pseudoprotease *csp*A detected amino acid cogerminants [38,63]. Bile acids (CA) induce c-di-AMP degradation in *C. difficile*, which derepresses genes encoding a solute import system protecting from hyperosmotic stress and bile salts [37]. Bile acids also induce damage to Gram-negative bacteria. CDCA and DCA induce reactive oxygen species (ROS)-mediated nucleic acid damage in *E. coli* and activate the SOS response [64]. Ox bile at as high as 20% induced nucleotide substitutions, frameshifts, and chromosomal rearrangements in *Salmonella enterica* [65]. There is little insight into the molecular mechanism of bile acids in inhibiting *C. perfringens* growth. In our study, we found that *C. perfringens* mutants were resistant to growth inhibition by DCA/isoalloLCA, suggesting the involvement of certain molecular signaling pathways. We are working on finding the mutated genes in the three mutants.

Because *C. perfringens* is a pathogen, we also investigate its virulence changes by the potent bile acids DCA and isoalloLCA. The reduction of toxin gene expression of *plc* and *cpe* by DCA was consistent with our studies that DCA reduces *C. perfringens* sporulation [16] and its induction of chicken NE [45]. Consistently, the *cpe* regulatory gene *spoOA* [20] was reduced by DCA. Interestingly, DCA also reduced the regulatory RNA gene *virT* expression. Because isoalloLCA was more potent in reducing *C. perfringens* virulence, it would be reasonable to argue that isoalloLCA would be more potent in reducing chicken NE. Besides modulating *C. difficile* virulence mentioned above, bile acids at 1% induce BrtA-mediated transcription of the multidrug efflux pump genes *mdrM* and *mdrT* in Gram-positive *Listeria monocytogenes* [66]. Bile acids also reduce the virulence in Gram-negative bacteria but not growth inhibition at physiology concentration. DCA or CDCA, but not other bile acids or detergents, enhances *Shigella* spp. adherence and invasion of HeLa cells [67]. CA at 0.5% and DCA at 0.1% represses *S. enterica* invasion through postranscriptionally destabilizing the transcription factor HilD [68]. Although bile (59% DCA and 59% CA) does not change *Shigella flexneri* growth, it induces *spE1/ospE2* gene expression and adherence to HeLa cell [69]. DCA modulates the PhoP-PhoQ two-component regulatory system in *Salmonella* spp. [70]. Ox bile at 3% inhibits the gene expression of the *S. enterica* pathogenicity island 1 (SPI-1) [71]. Ox bile at 0.2% reduces the gene expression of *ctxAB* (encoding choleratoxin) and of *tcpA* in *Vibrio cholerae* but increases bacterial motility [72]. Bile at 0.05% binds VtrC and induces *Vibrio parahaemolyticus* virulence type III secretion system 2 (T3SS2) through VtrA/VtrC activating VtrB [73]. DCA at 0.1% increases the gene expression of *ciaB*, *cmeABC*, *dccR*, and *tlyA* in *Campylobacter jejuni* [74]. BACTO bile salts at 0.8% reduces the expression of forty-one genes of enterocyte effacement (LEE) pathogenicity island in *Escherichia coli* O157:H7 but increases the expression of seventeen genes related to iron scavenging and metabolism [75]. Although the bacterial virulence gene expression in response to bile acids is complex due to various bacteria and bile, the accumulated data would generate consensus molecular mechanisms on the interaction of bacteria and bile in the future.

The other important findings in this study are the reduction of *asrA1* gene expression and H_2_S gas generation in *C. perfringens* by DCA and isoalloLCA. Consistently, DCA reduced the foul smell and ballooned small intestines in NE birds [45]. H_2_S is a foul-smelling gas [76]. H_2_S produced by animals and bacteria has been found to have pleiotropic effects on the physiology of organisms since its discovery. The ileums of rabbits, guinea pigs, and rats are relaxed by H_2_S donors [77]. H_2_S induces intestinal relaxation through potassium (K) channels, particularly apamin-sensitive small conductance calcium-activated potassium (SK) channels and glybenclamide-sensitive K (ATP) channels [78]. H_2_S inhibits both L-type calcium channels and BK_Ca_ channels in smooth muscle cells of rat colon [79]. There are various other signaling pathways in H_2_S-mediated motility and relaxation [24]. Hence, *C. perfringens*-produced H_2_S may relax and enlarge the intestine, slow digesta movement, and increase bacterial overgrowth, leading to exacerbated NE. It remains unknown whether H_2_S plays any role in the chicken NE intestinal inflammation, showing ischemia-perfusion-like histopathology [45]. The role of H_2_S in the intestinal inflammation of other animals is complex and, at times, contradictory. Fecal sulfide levels are increased in patients with ulcerative colitis, and effective treatment of relapsed inflammation is associated with a reduction in sulfide levels [80,81]. The pro-inflammatory effect of a high dose of H_2_S is shown in an LPS-induced model of endotoxic shock in male Swiss mice, where DL-propargylglycine (H_2_S synthesis enzyme inhibitor) reduces this effect [82]. Evidence against H_2_S having a causative role in ulcerative colitis comes from a study that fails to demonstrate elevated fecal sulfide levels in the disease state [83]. Exogenous H_2_S has a protective role in models of intestinal ischemia [84]. Both endogenous and exogenous H_2_S protect against nonsteroidal anti-inflammatory drug (NSAID)-induced gastritis [85]. H_2_S resolves inflammation by reducing leukocyte migration to the site of injury [86] and by enhancing neutrophil apoptosis [87]. Interestingly, *C. perfringens* α-toxin CPA blocks leukocyte migration and induces neutrophil necrosis [1]. It would be interesting to investigate the histopathology and immunity alterations in the presence of H_2_S and CPA.

Besides impacting the host, an increasing number of studies have established H_2_S gas as a major cytoprotectant and redox modulator in microorganisms. H_2_S influences bacteria activities, such as protecting bacteria from antibiotics and their induction of ROS oxidative stress [88]. The presence of plasmid-borne genetic elements enhances both basal H_2_S production and antibiotic resistance in multidrug-resistant strains of *E. coli* [89]. Cystathionine γ-lyase (CSE) generates H_2_S in two human pathogens, *Staphylococcus aureus* and *Pseudomonas aeruginosa*. CSE inhibitors reduce bacterial H_2_S biogenesis and suppress bacterial tolerance by disrupting biofilm formation and substantially reducing the number of persister bacteria that survive antibiotic treatment [90]. It remains elusive how H_2_S impacts *C. perfringens* growth, survival, and virulence.

Together, these results suggest that bile acids are signaling molecules regulating *C. perfringens* growth, H_2_S production, and virulence gene expression, with DCA and isoalloLCA as the most potent ones. The bile reduced *C. perfringens* virulence through transcriptionally modulating the pathogen signaling pathways. The findings in this study could be used to design new strategies to prevent and treat *C. perfringens*-induced diseases.

## Figures and Tables

**Table 1:** Primers used for the detection of virulent genes.

Gene	Primer Name	Sequence
*gyrA*	*gyrA*-F	TGCTTGTTGAC-GGACATGGT
	*gyrA*-R	ACAACTGGTTCTTTTTCCTCACC
*asrA1*	*asrA1*-F	ACATCTTCCACATCCTACACACA
	*asrA1*-R	TGATGAAATGGCTGGTGGACA
*plc*	*plc*- F	TGACACAGGGGAATCACAAA
	*plc*- R	CGCTATCAACGGCAGTAACA
*spoOA*	*spoOA*- F	ACAGGAATTGCAAAGGATGG
	*spoOA*- R	TTTTGTCTTGTCCAACAGCAG
*virT*	*virT*- F	TGAAATTGTTCTTTTGGATGAAGA
	*virT*- R	GCTTGAAAAGCTCCTGCCTA
*cpe*	*cpe*- F	CAACTGCTGGTCCAAATGAA
	*cpe*- R	GCATCTTTCGCCAGTTTCAA

## Data Availability

The data are presented in this paper.
